# Actively Open-Minded Thinking and Its Measurement

**DOI:** 10.3390/jintelligence11020027

**Published:** 2023-01-28

**Authors:** Keith E. Stanovich, Maggie E. Toplak

**Affiliations:** 1 Department of Applied Psychology and Human Development, University of Toronto, 252 Bloor St. West, Toronto, ON M5S 1V6, Canada; 2 Department of Psychology, York University, 126 Behavioural Science Building, 4700 Keele St., Toronto, ON M3J 1P3, Canada

**Keywords:** actively open-minded thinking, myside thinking

## Abstract

Actively open-minded thinking (AOT) is measured by items that tap the willingness to consider alternative opinions, sensitivity to evidence contradictory to current beliefs, the willingness to postpone closure, and reflective thought. AOT scales are strong predictors of performance on heuristics and biases tasks and of the avoidance of reasoning traps such as superstitious thinking and belief in conspiracy theories. Nevertheless, AOT is most commonly measured with questionnaires rather than performance indicators. Questionnaire contamination becomes even more of a danger as the AOT concept is expanded into new areas such as the study of fake news, misinformation, ideology, and civic attitudes. We review our 25-year history of studying the AOT concept and developing our own AOT scale. We present a 13-item scale that both is brief and accommodates many previous criticisms and refinements. We include a discussion of why AOT scales are such good predictors of performance on heuristics and biases tasks. We conclude that it is because such scales tap important processes of cognitive decoupling and decontextualization that modernity increasingly requires. We conclude by discussing the paradox that although AOT scales are potent predictors of performance on most rational thinking tasks, they do not predict the avoidance of myside thinking, even though it is virtually the quintessence of the AOT concept.

## 1. Actively Open-Minded Thinking and Its Measurement

In many previous publications we have articulated the differences between the concept of rationality and the concept of intelligence (Stanovich 2009, 2011; Stanovich et al. 2016). For our actions to be instrumentally rational, they must be the best means toward our goals, and for our beliefs to be epistemically rational, they must correspond to the way the world is—they must be true. Many components of instrumental rationality and epistemic rationality are assessed by heuristics and biases tasks in the psychological literature (Baron 2008; Evans 2014; Kahneman 2011; Kelman 2011; Koehler and Harvey 2004; Manktelow 2012; Stanovich 2004, 2011). Although there are several broader conceptualizations of rationality (Mele and Rawling 2004; Stanovich 2013), the so-called axiomatic approach to rationality (Luce and Raiffa 1957; Savage 1954)—which defines rationality as adherence to certain types of consistency and coherence relationships—is well-covered by the heuristics and biases literature.

Intelligence is an underlying component that facilitates performance on heuristics and biases tasks, but it is not the only one. Individual differences in thinking dispositions also underlie differences in rational responding. Thus, rationality is the more encompassing construct. The distinction between cognitive capacity (intelligence) and thinking dispositions is an old one in psychology. Cognitive capacities are the types of cognitive processes studied by information processing researchers seeking the underlying cognitive basis of performance on IQ tests. Perceptual speed, discrimination accuracy, working memory capacity, and the efficiency of the retrieval of information stored in long-term memory are examples of cognitive capacities that underlie traditional psychometric intelligence and that have been extensively investigated. Thinking dispositions, in contrast, are better viewed as cognitive styles. 

*Rational* thinking dispositions are those that relate specifically to the adequacy of belief formation and decision making—for example, the tendency to collect information before making up one’s mind, the tendency to seek various points of view before coming to a conclusion, the disposition to think extensively about a problem before responding, the tendency to calibrate the degree of strength of one’s opinion to the degree of evidence available, the tendency to think about future consequences before taking action, the tendency to explicitly weigh pluses and minuses of situations before making a decision, and the tendency to seek nuance and avoid absolutism. In short, individual differences in rational thinking dispositions include variation in people’s goal management, epistemic values, and epistemic self-regulation—differences in the operation of the reflective mind in our tripartite model (Stanovich 2011; Stanovich et al. 2016). 

On our Comprehensive Assessment of Rational Thinking instrument (CART; Stanovich et al. 2016), thinking disposition scales are supplemental to the subtests of the CART. Thinking dispositions are not primary measures of rationality themselves, because they are not maximizing concepts like the other constructs on the CART^1^. The most important thinking disposition that we measure on the CART is actively open-minded thinking (AOT).

## 2. Twenty-Five Years Trying to Measure AOT

Our research group has spent over 25 years trying to understand the AOT concept and attempting to measure it. We were originally inspired by the writings of Baron (1985, 1988, 1993), who first named and discussed AOT as an important thinking disposition (see Baron et al. 2023, for a history of the concept). In Stanovich and West (1997), we conceptualized AOT as a thinking disposition encompassing the cultivation of reflectiveness rather than impulsivity; the desire to act for good reasons; tolerance for ambiguity combined with a willingness to postpone closure; and the seeking and processing of information that disconfirms one’s beliefs. The items on the initial version (Stanovich and West 1997) of our AOT scale tapped reasoning styles such as the disposition toward reflectivity using items such as “If I think longer about a problem I will be more likely to solve it,” and “Intuition is the best guide in making decisions,” (the latter reverse-scored). Other items tapped willingness to consider evidence contradictory to beliefs (e.g., “People should always take into consideration evidence that goes against their beliefs”) and the willingness to consider alternative opinions and explanations (“A person should always consider new possibilities”). Some items tapped the willingness to postpone closure (“There is nothing wrong with being undecided about many issues”). Philosophically, the original scale focused strongly on issues of epistemic self-regulation raised in philosophical discussions (Goldman 1986; Harman 1995; Nozick 1993; Samuelson and Church 2015). The scale was a marker for the avoidance of epistemological absolutism; willingness to perspective-switch; and the tendency to consider alternative opinions and evidence.

In this paper, we will focus on the links between AOT and performance on heuristics and biases tasks from the reasoning literature, because that is where the bulk of our empirical offers have been directed. Recently, however, there has been a burgeoning literature (e.g., Ackerman and Thompson 2017; De Neys 2023) examining the meta-reasoning processes that are implicated when solving these types of reasoning tasks. Although we have not investigated connections between AOT and these meta-reasoning processes, there would seem to be a good deal of conceptual overlap. As mentioned above, Baron’s (1985, 1988) early conceptualization of AOT and our earliest attempts to operationalize the concept focused heavily on epistemic self-regulation. This emphasis provides a possible link to the meta-reasoning literature (Ackerman and Thompson 2017), where the issues of monitoring ongoing thinking, allocating cognitive resources, and attentional switching are paramount. The concept of cognitive decoupling, which we elaborate later in this paper, provides a potential connection between the extant literature on AOT and the growing literature on how meta-reasoning contributes to task outcomes (e.g., Ackerman and Thompson 2017; Raoelison et al. 2020; Thompson et al. 2013).

We have been investigating actively open-minded thinking for over two decades now and have been continually refining the scale since that initial study (Sá et al. 1999; Stanovich and West 2007; Stanovich et al. 2016; Stanovich and Toplak 2019). For example, Sá et al. (1999) introduced nine new items into the scale in order to measure an aspect of AOT that we termed belief identification. These items were inspired by a theoretical paper by Cederblom (1989) in which he argued for a potential thinking style centered around the extent to which people identify their beliefs with their concept of self (e.g., “Certain beliefs are just too important to abandon no matter how good a case can be made against them” [reverse-scored]). Other additions and subtractions of components occurred over the next decade. By 2007 (Stanovich and West 2007), we had a 41-item instrument that was subsequently trimmed down to 30 items in our CART for adults (Stanovich et al. 2016; 16 items in the short form).

In our initial studies (Stanovich and West 1997) we found that our AOT scale was moderately associated with the ability to evaluate arguments. This association held even when the variance due to cognitive ability was partialled out. In several subsequent studies, we found that our AOT scale predicted performance on a variety of heuristics and biases tasks, often even after partialling cognitive ability (Stanovich and West 1997, 1998a, 1998b, 2000). This performance pattern has been found in a variety of studies conducted in many labs and has been obtained across a plethora of heuristics and biases tasks, including noncausal base-rate tasks, hypothesis evaluation tasks, four-card selection tasks, covariation detection, gambler’s fallacy, conjunction fallacy, Bayesian reasoning, framing problems, ratio bias, sample size problems, and probability matching (Bruine de Bruin et al. 2007; Erceg et al. 2022; Finucane and Gullion 2010; Kokis et al. 2002; Parker and Fischhoff 2005; Pennycook et al. 2014; Sa and Stanovich 2001; Sá et al. 2005; Stanovich et al. 2016; Toplak et al. 2007; Toplak and Stanovich 2002; Toplak et al. 2011, 2014a, 2014b; Viator et al. 2020; Weller et al. 2018; West et al. 2008).

It is startling that a questionnaire measure tapping a thinking disposition correlates with so many heuristics and biases tasks and that it is often a unique predictor after cognitive ability is partialled. Equally impressive has been the recent expansion of the AOT concept as an explanatory mechanism in new and diverse areas (Baron 2019; Baron et al. 2015; Baron et al. 2023; Baron et al. 2017), including linking the concept to optimal information acquisition (Haran et al. 2013) and cognitive inhibition skills (Campitelli and Gerrans 2014).

One of the earliest linkages that we observed when we began studying the AOT was that it predicted pseudoscientific beliefs and superstitious thinking. It has consistently correlated with the presence of what we termed contaminated mindware—declarative knowledge that is incorrect and that leads to suboptimal action (see Rizeq et al. 2021; Stanovich 2011; Stanovich et al. 2016). For example, in one of our earliest studies (Stanovich and West 1997), several of the subcomponents of our first AOT test displayed significant negative correlations with superstitious thinking (flexible thinking = −.26; absolutism = −.23; dogmatism = −.19; categorical thinking = −.28). A later study using an updated composite AOT scale found a −.38 correlation between it and superstitious thinking (Toplak et al. 2011). Using a more refined AOT scale, we found a −.44 correlation with a university sample and −.59 with an mTurk sample (Stanovich et al. 2016), as well as correlations ranging from −. 39 to −.53 with Prolific samples (Stanovich and Toplak 2019). These findings have been much replicated, as AOT scales have been found to correlate with a variety of different measures of superstitious thinking and belief in the paranormal (Erceg et al. 2022; Jastrzębski and Chuderski 2022; Pennycook et al. 2020; Rizeq et al. 2021; Svedholm and Lindeman 2013; Svedholm-Hakkinen and Lindeman 2018). 

Conspiracy belief scales measure another form of contaminated mindware that has been linked to actively open-minded thinking. These beliefs, given the lack of evidence for them, appear to be remarkably prevalent (Oliver and Wood 2014), and they seem to be part of a cluster of thinking styles that interconnect with superstitious behavior and animistic thinking (Oliver and Wood 2014; Rizeq et al. 2021; Stanovich et al. 2016). 

Belief in specific conspiracy theories and general conspiratorial ideation have been found to correlate with AOT scales, but at a somewhat lower level than the correlations obtained with superstitious thinking. For example, Swami et al. (2014) found a significant negative correlation between the Stanovich and West (2007) AOT scale and the Belief in Conspiracy Theories Inventory (Swami et al. 2010, 2011), but the magnitude of the correlation was only −.07. Somewhat stronger results were obtained with the AOT scale that was part of our Comprehensive Assessment of Rational Thinking (Stanovich et al. 2016) and our Conspiracy Beliefs subscale. We found correlations of −.34 and −.26 in university samples but a higher −.48 correlation in an mTurk sample (Stanovich et al. 2016). Using a different AOT scale, we found correlations ranging from −.19 to −.29 in Prolific samples (Stanovich and Toplak 2019). These findings have been much replicated, as AOT scales have been found to correlate with a variety of different measures of conspiracy belief (Binnendyk and Pennycook 2022; Erceg et al. 2022; Jastrzębski and Chuderski 2022; Pennycook et al. 2020; Rizeq et al. 2021; Yelbuz et al. 2022).

## 3. Examining a Wider Range of Correlates Leads to New Questions about AOT Scale Composition

Recent expansion of the use of the AOT scale into areas such as belief in evolution (Deniz et al. 2008; Sinatra et al. 2003); skeptical processing of fake news (Bronstein et al. 2019); accuracy in future forecasting (Mellers et al. 2015); moral reasoning (Baron et al. 2015); religiosity/ideology (Baron 2019; Stanovich and Toplak 2019); and skeptical attitudes toward alternative medicine (Svedholm-Hakkinen and Lindeman 2018) has raised new questions about the specific compositional structure of the AOT scales in use. 

In Stanovich and Toplak (2019), we described how we were led to re-examine the way in which our original scale was constructed and revised over the years by reading some recent studies that inserted the AOT concept into discussions of religion and ideology. The extremity of the results of these studies gave us pause and forced us to think more about the logic of some of the items. First, there were the startling results of Piazza and Landy (2013), who reported some extremely high correlations between an AOT scale and various measures of religiosity: −.58 with an attitudes toward religion scale, −.59 with a religious faith questionnaire, −.63 with a Christian orthodoxy scale, −.58 with self-reported religiosity, and a truly astonishing correlation of −.70 with a morality founded on divine authority scale. Baron et al. (2015) observed a correlation similar to those of Piazza and Landy (−.61) between an AOT measure and a four-item religiosity scale. Likewise, Bronstein et al. (2019) reported a similarly high correlation of −.67 between a short-form AOT scale and a religious fundamentalism measure. Other studies, such as that of Yilmaz and Saribay (2017), have found similarly strong correlations (−.47) between AOT scores and social conservativism. 

We were startled by these high correlations (in the −.50 to −.70 range), because we have run over a dozen studies employing versions of AOT scales (see Stanovich et al. 2016) in which religiosity and political ideology have been included as demographics questions. We have very consistently found correlations in the much lower range of −.25 to −.40 between religiosity and the AOT (correlations with ideology are almost always even lower). When puzzling over the cause of these discrepancies in the association between religiosity and AOT that our group observes versus those reported in these other studies, one of the first things we noticed was that most of the other research tended to use short-form AOT scales—often short forms of fewer than 10 items. These are much smaller scales than the 41-item AOT measure that we were using over a decade ago (Stanovich and West 2007) and the 30-item revised measure that we used in the CART (Stanovich et al. 2016). 

More important than the sheer number of items, of course, is the specific composition of the short forms. Here, a deeper analysis of the items used across various studies revealed a potential source of the discrepancies between the results. That source appears to reside primarily in the items that Sá, West, and Stanovich introduced into the scale in 1999. Sá et al. (1999) termed these items belief identification items, but Stanovich and Toplak (2019) suggested the broader term belief revision items. Nine of these items were introduced into the AOT scale in our 1999 study. One item of a similar type was already in the earlier scale. Thus, the 41-item scale used in the mid-2000s by Stanovich and West (2007) consisted of 10 belief revision items out of a total of 41 (24.4%). The 30-item updated AOT subtest in our CART (Stanovich et al. 2016) had nine belief identification items (30% of the total). It was immediately of concern to us in perusing the short forms used in other studies that the proportion of belief revision items was substantially higher. Piazza and Landy (2013), in the study that obtained extremely high correlations with a host of variables measuring religiosity, used a seven-item AOT scale that contained five belief revision items (Yilmaz and Saribay 2017, used the same seven-item short form). Baron et al. (2015) used an eight-item AOT short form that contained four belief revision items. Bronstein et al. (2019), who reported a substantial correlation of −.67 with a religious fundamentalism measure, used an eight-item AOT short form that contained five belief revision items. Thus, the three studies displaying religiosity correlations of .55 or above used short forms of the AOT that were composed of 71.4%, 50%, and 62.5% belief identification items. This is much higher than the roughly 24–30% composition that we have used in our versions of this instrument. We conjectured that the high proportion of belief revision items in these other studies was the source of the high correlations with religiosity.

What is the feature of the belief revision items that might be augmenting correlations with religiosity? Consider two such items:

Beliefs should always be revised in response to new information or evidence.
One should disregard evidence that conflicts with your established beliefs. (reverse-scored)

The general thrust of this kind of item is that the subject is being asked what should be done when encountering evidence that conflicts with a prior belief or opinion. It is important to note that no *specific* prior opinion is mentioned in any of these items. It is just the generic word “belief” that is used. Of course, adjusting a prior belief based on new contradictory evidence is more or less easy to do depending upon what the belief is. For example, is it my belief that I voted the right way in the presidential election of 2020? Or is it my belief that the deli counter is better at Albertsons than at Safeway? The latter is obviously going to be a belief that is more easily conditioned by evidence than the former. The generic nature of the word “belief” in these items allows the respondent to insert any imaginary opinion as the belief in question. Potential social desirability considerations may lead most people to insert a belief that is easy to change. Thus, these items are almost inviting someone to fall prey to the bias blind spot—that is, thinking that others are characterized by a particular bias but that you yourself are not (Pronin 2007). 

All of the above might be true for a secular person, but a person with strongly held religious convictions might well be prone to see the word “belief” as referring to their spiritual beliefs—a class of beliefs that are not going to be easily altered by evidence. In contrast, a secular person might be much less likely to see the word “belief” as denoting an imaginary opinion that is so strongly held. Our conjecture was that what a religious person does when seeing the generic word “belief” is simulate an *actual* stance (their spirituality) that is much more difficult to reconsider based on evidence than a generic belief or an anodyne one.

To see this, one might imagine a secular person who answered one of our belief revision items affirmatively. To such a person who answered by saying, “Well of course I’d change my belief if I got contradictory evidence, that’s what an intelligent person does”, we might imagine the conversation continuing. “OK”, we might reply, “now imagine the belief is your vote against Trump in 2020. Would you be likely to change that based on new information?” It is doubtful that the item would be so enthusiastically endorsed if we substituted in the *specific* belief “my vote against Trump was a good thing”. 

To the extent that secular people are inserting an anodyne belief such as the preference for Pepsi over Coke as opposed to a belief strongly related to worldview such as belief in God or a particular religion, then they are advantaged on such items. This advantage, along with the corresponding disadvantage to the religious respondent who might slot in “belief in God” for the term “belief”, inflates the negative correlation between AOT and religiosity—such items are harder to agree to on the part of the religious-minded. This non-equivalence never occurred to us at the time we were creating the belief revision items, perhaps because of our own secular biases. No doubt, if the correlations had come out in the other direction, we would have been quicker to notice a problem, since those of us constructing the items were all secularists.

Stanovich and Toplak (2019) showed in a post hoc analysis of data from the CART (Stanovich et al. 2016) that belief revision items on the AOT subtest showed higher correlations with both religiosity and ideology than non-belief revision items did. Importantly, the nonbelief revision items show just as strong correlations with other variables such as superstitious thinking and belief in conspiracy theories. In a new experiment, we demonstrated that subjects high in religiosity are differentially affected by the word “belief” in an item and that using that term inflates correlations between AOT and religiosity—and, to a lesser extent, the correlation between AOT and ideology. Pennycook et al. (2020) reported converging findings, in that they found that a version of the AOT that substituted the word opinion for the word belief reduced the correlation between the AOT and religious beliefs from −.42 to −.20. The correlations with ideology and various social opinions were likewise reduced.

Assessing whether opinions and beliefs are flexibly conditioned by evidence is an important component of actively open-minded thinking, but using the word belief misleadingly inflates correlations in studies where the focus shifts to the larger set of issues that we mentioned above (the relation between AOT and religiosity, ideology, voting behavior, etc.). There is no doubt that a correlation between AOT and religiosity exists. It is just that it is in the range of −.20 to −.30 rather than −.65. The difference matters, because of the contexts in which many of the correlations in the range of −.60 to −.70 have been obtained. That context has been, in many cases, studies that have used only short forms of the AOT with modest reliabilities. If these −.65 to −.70 correlations were corrected for attenuation—or if the two variables were measured as latent constructs—it would not be surprising if the relationship between them approached −1.0. 

With individual differences in AOT entirely explained by religiosity, psychological research would then be saying to the public that religiosity and failing to think in an open-minded manner were, for all intents and purposes, the same thing—that being highly religious is virtually synonymous with being close-minded. Our findings, of course, support the weaker conclusion that there is a replicable moderate correlation between actively open-minded thinking and religiosity. 

It is increasingly the case that the psychological correlates of worldview, voting behavior, and ideological orientation are becoming points of contention in our divided political culture (Baron 2019; Baron et al. 2023; Crawford and Jussim 2018; Ditto et al. 2019; Duarte et al. 2015; Kahan et al. 2017; Stanovich 2017, 2021). If psychological studies of this type are increasingly becoming an adjunct of politics, it is important that psychology maintain its credibility as a neutral arbiter—a credibility that has been vastly eroded in recent years by empirical evidence of the ideological bias in our science (Ceci and Williams 2018; Crawford and Jussim 2018; Duarte et al. 2015; Ellis 2020; Haidt 2022; Jussim 2019, 2022; Stanovich 2021). Greater intellectual diversity in our own lab years ago might have prevented us from continuing to use items in our AOT scale that inflated negative correlations with religiosity.

## 4. Scale Structure Changing over Time

Discussing the history of the belief revision items opens up the topic of the composition of AOT scales and their evolution over time. At the very beginning of our studies of AOT, we included in our scale items that we constructed ourselves that were designed to tap the AOT concept, but we also included a variety of items that were included on scales tapping related constructs. For example, in our initial 1997 scale (Stanovich and West 1997), we constructed items tapping the disposition toward reflectivity (“If I think longer about a problem I will be more likely to solve it”; “Difficulties can usually be overcome by thinking about the problem, rather than through waiting for good fortune”; “Intuition is the best guide in making decisions”; and “Coming to decisions quickly is a sign of wisdom”, the latter two reverse-scored), willingness to consider evidence contradictory to beliefs (e.g., “People should always take into consideration evidence that goes against their beliefs”), willingness to consider alternative opinions and explanations (“A person should always consider new possibilities”, “Considering too many different opinions often leads to bad decisions”, the latter reverse-scored), and tolerance for ambiguity combined with a willingness to postpone closure (“There is nothing wrong with being undecided about many issues”, “Changing your mind is a sign of weakness”, and “Basically, I know everything I need to know about the important things in life”, the latter two reverse-scored).

However, to these kinds of items, we added items from extant scales of dogmatism, the openness–values and openness–ideas facets from the Revised NEO Personality Inventory (Costa and McCrae 1992), and scales measuring absolutism and categorical thinking (Epstein and Meier 1989). As discussed above, nine belief revision items were added in 1999 (Sá et al. 1999). Our 41-item scale published in Stanovich and West (2007) has been much used, although it was still, like our earlier scales, quite a conceptual amalgam.

The 30-item AOT scale that we published in the CART, however, represented a more conceptually coherent concept of AOT than our earlier measures. Items from many scales where the construct was related to AOT but not central to the AOT concept were eliminated. So, for instance, items tapping dogmatism, absolutism, and categorical thinking were eliminated, because although these concepts are related to (lack of) AOT, they are not AOT’s defining features. Newton et al. (2023), for example, found that dogmatism/categorical thinking was separable from AOT. The openness–ideas facet items from the Revised NEO Personality Inventory were removed because they were less conceptually related to AOT than they were to need for cognition (e.g., “I enjoy working on ‘mind-twister’-type puzzles”).

We removed the openness–values facet items from the Revised NEO Personality Inventory that were in our 1997 scale for a different reason. Some of these items are contaminated by ideological and/or anti-religious bias. Specifically, they require high scorers to have a progressive worldview—high scores are harder to achieve for conservatives or those higher in religiosity (Charney 2015). For example, the purpose of the item “I believe that we should look to our religious authorities for decisions on moral issues” is clearly to probe whether the individual is prone to rely on authorities to determine moral beliefs. But the specific authority that the subject has to ignore in order to score highly is a religious authority. There is no corresponding item testing whether the subject is equally reliant on secular authorities (see Stanovich and Toplak 2019 for the importance of content in an item like this). Is it more close-minded to rely on a theologian for moral guidance than to rely on a university “bio-ethicist?” Secular subjects are guaranteed to score higher on an item like this, which virtually builds in a correlation between openness and religiosity. Another item—“I believe that the different ideas of right and wrong that people in other societies have may be right for them”—seems to require that full-blown cultural relativism be endorsed in order to receive a high openness score. However, the only ideological niche where such strong relativism is highly endorsed is in an extreme form of multiculturalism that exists only on the political left. Thus, we removed all of the openness–values items from the CART version of the AOT to avoid building ideology into this scale^2^. If there is a correlation between AOT and liberalism, we want that to be an actual fact about human psychology and not something that is an artifact of item construction.

In addition to the removal of these classes of items, the 30-item CART version of the scale contained some new items that were taken from the MRM scale introduced by Stanovich (2008). MRM refers to the master rationality motive proposed by Stanovich (2008). That motive is the desire to act in accordance with good reasons. Items from this scale were added to the AOT scale on the CART, including items such as “I like to think that my actions are motivated by sound reasons” and “If a belief suits me then I am comfortable, it really doesn’t matter if the belief is true (reverse scored)”.

## 5. Toward a New 13-Item Recommended AOT Scale

All of these changes made the 30-item CART version of our AOT scale a much more coherent measure than the much-used 41-item version in Stanovich and West (2007), probably similar in coherence to humility scales^3^. This was even more true of the shortened 16-item version of the CART AOT scale. The greater coherence of the CART version was predictable from the factor analysis of the 41-item Stanovich and West (2007) version conducted by Svedholm-Hakkinen and Lindeman (2018). They argued that a four-factor solution was needed to capture the multidimensional nature of the scale. Interestingly, however, three of the four factors were composed of items from categories that we have since eliminated from the 30-item CART version of the scale. Consistent with our argument above, one of these three factors was labeled Liberalism and was composed largely of more openness–values items of the type that we have eliminated (“I believe that the different ideas of right and wrong that people in other societies have may be right for them”). Another factor was labeled dogmatism by Svedholm-Hakkinen and Lindeman (2018) and consisted of openness–values items that we have eliminated, in addition to dogmatism and categorical thinking items that have also been eliminated (“I think there are many wrong ways, but only one right way, to almost everything”). Finally, they identified a factor they called Belief Personification that also consisted of dogmatism items and categorical thinking items that we have eliminated (“I tend to classify people as either for me or against me”). 

In fact, Svedholm-Hakkinen and Lindeman (2018) identified just one factor from items we are still using. They labeled the factor Fact Resistance, but it was actually composed primarily of items that we have called belief revision items. Importantly, when they correlated the Stanovich and West (2007) scale with other criterion variables such as superstitious thinking and trust in alternative medicine, the belief revision factor was the primary correlate. Associations with the other three factors were quite low. Overall, the analysis of our 41-item scale by Svedholm-Hakkinen and Lindeman (2018) supports the revisions in the AOT scale that we have made in the CART (Stanovich et al. 2016).

It would be nice to conclude here that the AOT scale in the CART should be the default scale for researchers in this area. Unfortunately, that conclusion would be premature. First of all, at 30 items, the scale is overly long for many investigations with time limits. Even the 16-item shortened form is on the long end for some studies. Many investigations in the AOT literature use scales no longer than 10 items. 

Even more importantly, our AOT scale in the CART contains a number of belief revision items that implicate the ideological/religious bias that we discussed earlier. It is becoming increasingly important that AOT scales not contain biases of these types as the investigations using the scale proliferate into politically charged areas (e.g., studies of fake news, conspiracy theories, and politically charged issues such as climate change). Increasingly, the scale is being used to adjudicate issues in the literature of political psychology. It is even more important in these types of areas that the scale not be biased ideologically or in terms of worldview. The conclusion that “liberals are more open-minded” will be specious if based on an instrument that has such a correlation built-in.

For all of these reasons, we feel compelled to present a new recommended AOT scale based on our own research and our reading of the recent AOT literature. That scale is presented in Table 1. In the right-hand column of Table 1, we have listed the primary AOT concepts that are tapped by each item. Several of the items tap more than one AOT concept. Subjects responded on a six-point scale with no neutral point: disagree strongly (1), disagree moderately (2), disagree slightly (3), agree slightly (4), agree moderately (5), agree strongly (6).

There are 13 items in our recommended scale, 12 of which appeared in the AOT scale used in the CART. However, several of these items, particularly the belief revision items, have been slightly rewritten. For example, to reflect the findings of Stanovich and Toplak (2019), the word belief was removed from items—usually being replaced with the word “opinion” or “something I think” (see Pennycook et al. 2020, who used this substitution). The single item that did not appear in the AOT scale of the CART but is included in Table 1 as part of the recommended scale (“Intuition is the best guide in making decisions”, reverse-scored) was originally in the Stanovich and West (2007) scale, and thus, we have a lot of data on that item as well. Based on an analysis of the 12 items in Table 1 that were present in the CART AOT, this scale would have a reliability of .84 (Cronbach’s alpha) and would factor as having a single dominant factor (only one eigenvalue > 1).

## 6. The AOT Measures Psychological Tendencies to Decouple and Decontextualize—A Critical Aspect of Modernity

It is important to measure AOT carefully and without bias for two reasons. First, as noted above, the concept is increasingly being used in broader areas of sociocultural concern such as belief in alternative medical practices; belief in pseudoscience and conspiracies; detection of fake news; moral decision-making; and debates about the origins and correlates of political ideologies (Baron 2019; Bronstein et al. 2019; Pennycook et al. 2020; Stenhouse et al. 2018). Secondly, it is becoming increasingly clear that in the domain of rational thinking, AOT is a uniquely potent predictor. It is ubiquitously linked to subtests in our CART test (Stanovich et al. 2016). The CART is a very comprehensive measure of rational thought and is composed of 20 different subtests (and 4 supplemental scales, which include AOT). Our 30-item AOT scale not only was correlated with every one of the 20 subtests but accounted for variance over and above cognitive ability in the vast majority of them (17 out of 20). Despite the multifariousness of the rationality construct itself (which is why the CART contains 20 subtests), a particular thinking style—actively open-minded thinking—does permeate almost all of the components (from probabilistic reasoning to avoiding overconfidence and many more). 

What are the central features of thinking that make the AOT such a good predictor of rational thinking? We would argue that the common psychological dimension is the tendency to engage in cognitive decoupling (Stanovich 2011; Stanovich and Toplak 2012). To a lesser extent, the items may tap a related tendency toward the decontextualization of problems. 

Cognitive decoupling is particularly relevant to the heuristics and biases tasks that operationally define rationality in cognitive science (Baron 2008; Kelman 2011; Stanovich 1999, 2012), because these tasks often create hostile problem-solving environments (Stanovich et al. 2016). Heuristics and biases tasks are designed to trap the cognitive miser (Kahneman 2011; Stanovich 2004, 2018). Tasks from this literature often have an intuitively compelling wrong answer that must be overridden, as in the famous Linda conjunction fallacy problem (Tversky and Kahneman 1983), where even sophisticated responders are tempted by the attractiveness of the wrong answer. Stephen J. Gould’s (1991) introspection was that “I know the conjunction is least probable, yet a little homunculus in my head continues to jump up and down, shouting at me—‘but she can’t be a bank teller; read the description’” (p. 469).

In a probabilistic reasoning task from the heuristics and biases literature, the entire point is to see how dominant or nondominant the statistical interpretation is over the narrative interpretation. The fact that many heuristics and biases tasks can be construed by the subject in different ways (a statistical interpretation versus a narrative interpretation, for instance) is often seen as a weakness of such tasks, when in fact it is the design feature that makes the task diagnostic. 

As a result, heuristics and biases tasks create a more hostile reasoning environment than typical IQ test problems, in that the latter do not contain enticing lures toward an incorrect response. Neither is the construal of an intelligence test item left up to the subject. Instead, the instructions to an IQ test item attempt to remove ambiguity in a way that is not true of a heuristics and biases problem. The famous Linda conjunction problem would be a prime case in point. The instructions purposefully do not tell the subject how to weight the conflicting cues—the similarity of the description (“deeply concerned with issues of discrimination and social justice, and also participated in anti-nuclear demonstrations”) to the classification (“feminist bank teller”) and the subset/superset relationship between feminist bank teller and bank teller. Most subjects detect the two conflicting cues in the problem (De Neys 2014, 2023), but the instructions pragmatically obscure the fact that the correct weighting of the cues is 0/100.

Thus, rational thinking paradigms attempt to measure the *propensity* to use a cognitive skill in a way that IQ tests do not. People who can answer an explicit probability question on a test or can accurately define “control-group” when asked may not invoke these principles when their relevance to a problem is partially disguised.

Cognitive decoupling is implicated in such hostile task environments in two ways (Oaksford and Chater 2012; Stanovich 2011; Stanovich and Toplak 2012). It is implicated in the inhibitory override of the intuitive response triggered by many heuristics and biases tasks, but it is also implicated in the sustained simulation of alternative worlds that is necessary to compute the correct response. The first type of cognitive decoupling—inhibition of the prepotent response—is akin to that studied in the executive functioning literature (Kovacs and Conway 2016; Miyake and Friedman 2012; Nigg 2017). However, the ability to suppress miserly processing gets the job only half done. Suppressing one response is not helpful unless there is a better response available to substitute for it. Where do these better responses come from? One answer is that they can come from processes of hypothetical reasoning and cognitive simulation (Evans 2007, 2010; Evans and Stanovich 2013; Stanovich 2011). When we reason hypothetically, we create temporary models of the world and test out actions (or alternative causes) in that simulated world. Decoupling is necessary in order to prevent our representations of the real world from becoming confused with representations of imaginary situations. The tendency to initiate such decoupling for the purposes of simulation is a dispositional variable, separable from cognitive capacity (the ability to sustain the decoupling). 

Given this understanding of the importance of cognitive decoupling in heuristics and biases tasks^4^, our conjecture is that AOT scales tap the propensity to engage in these types of cognitive operations. For example, some AOT items relate to avoiding miserly processing and overriding the tendency to fix beliefs quickly or to decide quickly: “Coming to decisions quickly is a sign of wisdom” (reverse-scored). Others tap the willingness to consider possibilities beyond the focal model that is in the mind, e.g., “Considering too many different opinions often leads to muddled thinking” (reverse-scored), “Changing your mind is a sign of weakness” (reverse-scored), and “A person should always consider new information”. Additionally, many belief revision items require the subject to hold an existing belief in abeyance while simulating the effect of new information on the original belief—classic cognitive decoupling. All of these types of items are included in the recommended AOT scale in Table 1.

AOT scales capture global attitudes that make people more willing to decouple from strong default responses and consider new and/or conflicting evidence—or, for those responding on the other end of the scale, to be more comfortable with natural responses and accumulated knowledge. The tendency to be comfortable with responses that seem intuitive or that have been imbibed by repetition in familiar environments is probably the factor that accounts for the negative .20 to .30 correlations with religiosity that are observed in the literature in scales that are not overly contaminated with the biasing term “belief”.

The need to cognitively decouple is increasingly a requirement of modernity itself (see Stanovich 2004 for a more comprehensive discussion of this point). Modernity is the result of long historical trends that have replaced local/particular traditions with science and rationality as the arbiters of truth claims. This shift coincides with an increase in environments for thinking that are hostile rather than benign. IQ tests do not pick up these hostile aspects of the cognitive environment of modernity—but rational thinking tasks do. In fact, critics who charge that heuristics and biases tasks are artificial are missing an important point. The *kind* of “artificiality” that they display represents a strength of such tasks rather than a weakness. It is a design feature, not a bug. Years ago, Einhorn and Hogarth (1981) made the telling point that “in a rapidly changing world it is unclear what the relevant natural ecology will be. Thus, although the laboratory may be an unfamiliar environment, lack of ability to perform well in unfamiliar situations takes on added importance” (p. 82).

What Einhorn and Hogarth are pointing out is that the argument that laboratory tasks are not like “real life” is becoming less and less true. “Life”, in fact, *is becoming more like the tests!* For example, market economies contain agents who will exploit automatic responding for profit (better buy that “extended warranty” on a $150 electronic device!). This puts a premium on overriding intuitive responses that will be exploited by others in a market economy. The danger of such miserly tendencies (Stanovich 2018) in the domain of personal finance is suggested by the well-known finding that consumers of financial services often purchase high-cost products that underperform in terms of investment return when compared to the low-cost strategies recommended by true experts (e.g., dollar-cost averaging into no-load index mutual funds; see Bazerman 2001). The reason is, of course, that the high-cost fee-based products and services are the ones with high immediate recognizability in the marketplace. 

Many rational thinking tasks require subjects to decontextualize in a particular way—by “ignoring what they know” or by ignoring irrelevant context (belief bias in syllogisms, the famous Linda problem, etc.). That makes these tasks a good proxy for an aspect of scientific thinking, because, in science, we are often required to ignore what we know or believe. Testing a control group when you fully expect it to underperform compared to an experimental group is a form of ignoring what you believe. Science is a way of systematically ignoring what we know, at least temporarily (during the test), so that we can recalibrate our belief after the evidence is in. 

Likewise, many aspects of the contemporary legal system put a premium on detaching prior belief and world knowledge from the process of evidence evaluation. Modernity increasingly requires decontextualizing in the form of stripping away what we personally “know” due to its emphasis on such characteristics as fairness, rule-following despite context, even-handedness, sanctioning of nepotism, unbiasedness, universalism, inclusiveness, and legally mandated equal treatment.

## 7. The Paradox of AOT and Myside Thinking

A consistent observation in our earliest studies of individual differences in rational thinking was that almost every cognitive bias was correlated with intelligence as measured with a variety of cognitive ability indicators (Stanovich 1999). As discussed above, individual differences in most cognitive biases were also predicted by actively open-minded thinking.

Despite these consistent findings involving almost every other cognitive bias, myside bias has failed to correlate with AOT scales in the same manner as other biases. For example, in our study using Perkins’ (1985) argument generation paradigm (Toplak and Stanovich 2003), we found substantial myside biases on several issues (people tended to give more arguments in favor of their position than against), but the degree of myside bias was not correlated with several thinking dispositions, including AOT, dogmatism, and need for cognition. Macpherson and Stanovich (2007) examined myside bias in both argument generation and evidence evaluation and also measured two different thinking dispositions: AOT and need for cognition. None of the four resulting correlations were significant.

In our studies of naturalistic myside bias (Stanovich and West 2007) and argument evaluation (Stanovich and West 2008), relationships between myside bias and rational thinking dispositions were also negligible. Guay and Johnston (2022) examined myside thinking in political reasoning and found that the need for certainty and openness did not predict the magnitude of the myside effect.

Kahan and Corbin (2016) found an interaction between myside thinking and AOT scores, but the interaction was in the opposite of the expected direction. Conservatives and liberals who were high in AOT had more diverging opinions on climate change than conservatives and liberals who were low in AOT^5^. Stenhouse et al. (2018) found no significant interaction between AOT and ideological difference in climate-change attitudes. Although not replicating the interaction observed by Kahan and Corbin (2016), the Stenhouse et al. (2018) results (as well those of Clements and Munro 2021) converged with their results and those of Macpherson and Stanovich (2007) and Stanovich and West (2007) in finding no evidence that higher AOT scores attenuate tendencies toward myside thinking. 

In a followup study, Eichmeier and Stenhouse (2019) found a significant correlation between party identification and AOT scores (as have most studies; see Stanovich and Toplak 2019). However, using an argument evaluation paradigm, they found no indication that AOT scores were related to the myside bias observed in the argument strength ratings (see also Beatty and Thompson 2012; Clements and Munro 2021). Thus, the findings from the Stenhouse lab (Eichmeier and Stenhouse 2019; Stenhouse et al. 2018) are exactly parallel to those from the Stanovich lab (Macpherson and Stanovich 2007; Stanovich and Toplak 2019; Stanovich and West 2007). Both find that AOT scores correlate in the .20 to .30 range with ideology/partisanship, but neither lab finds an indication that AOT itself actually predicts the avoidance of myside bias. Although conservatives score lower on AOT scales, they do not display larger myside bias effects than liberals (Ditto et al. 2019; Guay and Johnston 2022; Stanovich 2021).

This convergence of findings is disconcerting, because of all the biases one would expect to be correlated with AOT, it would be myside bias (Baron 1993, 2019; Baron et al. 2023). Baron et al. (2015) argue that “AOT is a set of dispositions aimed at avoiding ‘myside bias’, the tendency to think in ways that strengthen whatever possible conclusions are already strong” (p. 267). In a later treatment of the concept, Baron et al. (2023) argue that the core of AOT encompasses avoiding myside bias and avoiding overconfidence in favored conclusions. The findings indicating that AOT does not correlate with direct measures of myside bias constitute an embarrassment to this view. The findings also do not fit well with our view, articulated above, that the AOT taps processes of decoupling and decontextualization that support the detachment needed to deal with the hostile environment of heuristics and biases tasks. It seems as though such detachment from the intuitive response is facilitated on normal heuristics and biases tasks such as the Linda problem but is not brought to bear when the problem involves detaching from a favored belief.

We would argue that strength of belief is an issue here that may make the findings a little more congenial to our view. *Belief bias* does indeed correlate with AOT as we would expect, but myside bias does not. Belief bias occurs when people have difficulty evaluating conclusions that conflict with what they know about the world. For example: “all living things need water; roses need water; therefore, roses are living things” is an invalid syllogism. Belief bias has been most extensively studied in the syllogistic reasoning and conditional reasoning literatures (Evans 2017), but it is observed in other paradigms as well (Levin et al. 1993; Stanovich and West 1997, 1998b; Thompson and Evans 2012).

Belief bias is not the same as myside bias. Belief bias occurs when real-world knowledge interferes with reasoning performance. Myside bias is a bias toward searching and interpreting evidence in a manner that tends to favor the hypothesis we want to be true (Mercier 2017; Stanovich et al. 2013). What turns a belief bias into a myside bias? Myside bias refers to processing in favor of existing opinions that are currently highly valued. To use a distinction discussed years ago by Abelson (1988), myside bias concerns the beliefs that individuals hold with high conviction. Convictions—unlike more typical beliefs—are accompanied by emotional commitment and ego preoccupation. Convictions also tend to have undergone more cognitive elaboration (see Abelson 1988; and also Fazio 2007, and Howe and Krosnick 2017, for more contemporary discussions). Skitka et al. (2005) found that attitudes rooted in moral mandates tended to become convictions. Convictions that were rooted in such moral judgments were especially potent predictors of outcome variables (social distance, good will, etc.). 

To illustrate the difference between a simple belief and a conviction, imagine a thought experiment where you were on another planet (Zircan), otherwise exactly like Earth, and heard from someone that on planet Zircan roses were never red and were always brown. You would have no trouble acquiring that belief. You would feel no urge to argue with anyone that roses can be red. On planet Zircan, they simply are not, and you would have no trouble giving up your belief that roses can be red. On the other hand, if you were to hear that on planet Zircan it was believed that left-handed people were morally inferior to right-handed people, you likely would not accept that belief and in fact would try to argue against it. You would instead defend your belief that the moral worth of human beings does not depend on whether they are left-handed or right-handed. That belief is a conviction for you in a way that the belief that roses can be red is not.

Convictions often derive from worldviews that spawn so-called protected values—those that resist trade-offs with other values (Baron and Spranca 1997). Protected values (sometimes termed sacred values; see Ditto et al. 2012; Tetlock 2003) are viewed as moral obligations that arise from deontological rules concerning action, and the thought of violating them often provokes anger. Experiments have shown that subjects are reluctant to trade or engage in monetary tradeoffs when protected values are at stake (Baron and Leshner 2000; Bartels and Medin 2007). Interestingly, Fisher and Keil (2014) found that the closer beliefs were to convictions, the more poorly calibrated subjects were—almost always believing that they could provide good arguments for their convictions when in fact they could not.

In further writings on the idea that some beliefs can become convictions, Abelson (1986; Abelson and Prentice 1989) makes the distinction between what he calls testable beliefs and distal beliefs. Testable beliefs are closely tied to the real world and the words we use to describe that world (e.g., roses are red). They can be verified by observations—sometimes easily made personal observations but other times requiring reliance on the expertise of others and the more sophisticated methods of science. In contrast, distal beliefs cannot be directly verified by experience, nor can they be easily confirmed by turning to experts or scientific consensus. For example, you may think that pharmaceutical companies make excessive profits, or that your state should spend more on mental health and less on green initiatives. Certainly, economic statistics and public policy facts might condition distal beliefs such as these (either strengthening or weakening our attachment to them) but they cannot verify our distal beliefs in the same manner that they can verify testable ones. Many distal beliefs embody our values. When they do, they are apt to become convictions, because they will lead to emotional commitment and ego preoccupation, as argued by Abelson (1988). Distal beliefs often derive from a person’s general worldview or, in politics, from their ideology.

Myside bias centers on distal beliefs, not testable ones. Belief bias, in contrast, concerns testable beliefs. This is why belief bias is more remediable by education and more correlated with cognitive ability than myside bias (Stanovich 2021). The proposition that health care spending is the second largest item in the US federal budget is a testable belief. The proposition that Americans spend too much on health care is a distal belief. Certainly, economic facts might alter our attitude toward the latter proposition, but they cannot verify this distal belief in the same manner that they can verify testable beliefs. The conclusions that interfere with reasoning in the case of belief bias are testable beliefs. Myside bias, in contrast, occurs when people evaluate and generate evidence in a manner favorable toward their prior opinions and attitudes—where the attitudes in question are convictions.

It is possible that these distinctions (testable versus distal; ego involvement versus noninvolvement; sacred values versus non-sacred) help to explain the curious paradox regarding AOT as a bias predictor—namely, that it predicts a plethora of biases except the one closest to its definition. Building on our view of AOT as a measure of the tendency to detach and decontextualize, one hypothesis might be that with myside bias paradigms, we are seeing the limits of individual detachment. Heuristics and biases tasks, as discussed above, often involve a conflict between a classically normative response and a classically nonnormative one^6^. De Neys (2014, 2023) has shown that in many cases the conflict between the two responses is detected at some cognitive level. The detected conflict might broach awareness to a sufficient degree that tendencies toward detachment can be helpful. In contrast, many myside bias paradigms (particularly the more naturalistic ones; see Stanovich and West 2007) may not provide opportunities for any conflict to be detected, thus neutering the possibility of high AOT subjects using their skills. Alternatively, the involvement of convictions may be overwhelming even in cases where awareness of alternative reactions has taken place.

Relevant here may be another property of myside bias—that it displays little domain generality (Stanovich 2021). People who display a high degree of myside bias in one domain do not tend to show a high degree of myside bias in a different domain. However, different beliefs vary reliably in the degree of myside bias that they engender. In short, it might not be people who are characterized by more or less myside bias but beliefs that differ in how strongly they are structured to repel contradictory ideas. These facts about myside bias have profound implications because they invert the way we think about beliefs. Models that focus on the properties of acquired beliefs, such as memetic theory, may provide better frameworks for the study of myside bias (see Stanovich 2021). The focus of memetics on the properties of beliefs rather than the psychological characteristics of the host is consistent with research showing that the degree of myside bias is better predicted by the former than the latter. They also might render the individual difference findings regarding AOT somewhat less paradoxical.

Detaching from a prepotent response in a heuristics and biases task such as the Linda problem may be vastly easier than using AOT tendencies toward detachment and decoupling to overturn a conviction and/or weaken a commitment to a sacred value. The levels of detachment and decontextualization required for the latter are orders of magnitude higher than the parallel cognitive requirements in a typical heuristics and biases task. This would be consistent with the argument previously made by Stanovich (2021) that myside bias is an outlier bias in the rational thinking literature. 

The parallel explanation from within Baron’s (2017; Baron 2019; Baron et al. 2015; Baron et al. 2023) conception of the AOT stresses that responses on the scale are an indication of the endorsement of norms or standards of good thinking. However, people adhere more or less to those standards, and they may differentially adhere to them based on the issue in question. Hence, Baron (2017) argues that “People may endorse AOT in a self-report questionnaire about beliefs, and they may behave consistently with it in most domains, but they may have gaps when they are strongly committed to a particular view” (p. 2). This argument is very parallel to that made with respect to our own decoupling/detachment view of what the AOT measures. Since most myside paradigms involve issues where the subjects are indeed likely to be “strongly committed to a particular view”, the Baron framework will arrive at the same place as us regarding individual differences in myside tasks that deal with conviction-based beliefs—AOT will not be a good indicator of who avoids myside bias in those domains. That means, however, that AOT will be less useful in some of the domains where we need it most—for example, social policy and politics.

For the first several decades of work in the heuristics and biases tradition (Kahneman and Tversky 1973; Tversky and Kahneman 1974), from the 1970s to the 1990s, myside bias was treated as simply another bias on a growing list of biases (anchoring bias, hindsight bias, availability bias, etc.) and its occurrence in the laboratory paradigms that were designed to study it was deemed non-normative, without much discussion in most papers. That initial stance now seems oversimplified. Myside bias does not act like any other bias in the traditional lists of thinking errors in this literature in terms of individual differences. We have also noted how it shows little domain generality. 

Finally, Stanovich (2021) has reviewed the extensive literature showing that it is not easy to demonstrate that myside bias is non-normative. It is unclear whether most myside thinking should even be considered a bias that leads to non-normative responses. For example, many utility-based theories that model beliefs in terms of cost and benefits (Loewenstein and Molnar 2018; Sharot et al. 2023) show that the early dismissal of myside bias as an irrational tendency was premature. Even in the domain of pure epistemic rationality, Stanovich (2021) has discussed how so-called knowledge projection models of myside bias show it is rational in many circumstances (Hahn and Harris 2014; Koehler 1993). The detachment and decoupling tendencies of AOT may not work against the epistemic mechanisms rationally acting as governors and damping down belief change. However, AOT does predict normative responding in many other tasks that do not have so many inertial components as myside bias.

## 8. Final Thoughts

At the end of a thoughtful chapter on AOT, Baron et al. (2023) mentioned that, in their view, “AOT is thus a moral virtue as well as a personal one” (p. 24). This statement provides a provocative way to probe our feelings about our own work. Specifically, do we believe that AOT is a moral virtue? Referring to AOT in the abstract, that is a difficult question for us, although we have some sympathy with the position. However, we have less sympathy with the idea that *extant* AOT scales are measuring something we would want to call a moral virtue. Undoubtedly, AOT scales are measuring something very central to the kind of thinking that is tapped on heuristics and biases tasks. Because such tasks tap aspects of rationality, AOT scales are measuring something very important. Nevertheless, the inability of AOT scales to associate with the avoidance of myside bias in a variety of paradigms is a very troubling finding. Avoiding myside bias is the quintessence of the most important theoretical treatments of actively open-minded thinking (Baron 1993, 2019; Baron et al. 2023). To say that we have a measure that predicts much other rational thinking but not the quintessence of the concept is a disconcerting conclusion. 

It is a disconcerting conclusion in just the way that the conclusion to Steve Pinker’s (2021) recent book on rationality was disconcerting. In the first nine chapters of his book, we learn that humans have many tools of rationality at their disposal. The brain is full of automatic propensities that have been honed over millennia to optimally regulate our responses to stimuli in environments that are not rapidly changing. Also available to us are all the cultural tools of rational thought that have been discovered throughout history (probabilistic reasoning, signal detection theory, expected utility theory, logic, game theory, scientific inference). By the process of cultural ratcheting, we have accomplished any number of supreme achievements such as curing illness, decoding the genome, and uncovering the most minute constituents of matter. All of this exists alongside surveys showing that 41% of the population believes in extrasensory perception, 32% in ghosts and spirits, and 25% in astrology—just a few of the pseudoscientific beliefs that Pinker (2021) mentions. These facts highlight what Pinker calls the rationality paradox: “How, then, can we understand this thing called rationality which would appear to be the birthright of our species yet is so frequently and flagrantly flouted?” (p. 6).

Pinker (2021) admits that the solution to this “pandemic of poppycock” (p. 286) is not to be found in correcting the many thinking biases that he covers in his book. We cannot remediate this kind of rational thinking through providing information. People captured by this poppycock have too much mindware—not too little (see Stanovich et al. 2016). Pinker notes that “nothing from the cognitive psychology lab could have predicted QAnon, nor are its adherents likely to be disabused by a tutorial in logic or probability” (p. 287). This admission uncomfortably calls to mind a quip by Scott Alexander (2021) that “of the fifty-odd biases discovered by Kahneman, Tversky, and their successors, forty-nine are cute quirks, and one is destroying civilization. This last one is confirmation bias—our tendency to interpret evidence as confirming our pre-existing beliefs instead of changing our minds.” This quip is not literally correct, because the “other 49” are not “cute quirks” with no implications in the real world. In his final chapter, Pinker describes and cites research showing that these biases have been linked to real-world outcomes in the financial, occupational, health, and legal domains (as we did in the last chapter of our book on the CART, Stanovich et al. 2016). They are not just cute quirks. Nevertheless, the joke hits home. That is why Stanovich (2021) wrote a whole book on the one bias that *is* “destroying civilization”—myside bias. 

Relationships involving the AOT parallel the Alexander quip—it predicts most of the “other 49” and fails to predict “the one that is destroying civilization”. It is clear why it is a bias that is difficult to avoid. It involves convictions—our most sacred and emotionally salient beliefs. To avoid it, people would need to be more skeptical of the strongest beliefs that they have acquired. They would have to learn to treat their beliefs less like possessions and more like contingent hypotheses (see Stanovich 2021). People would also need to be particularly skeptical of the beliefs that were acquired in their early lives—those that were passed on by parents, relatives, and their peers. It is likely that these beliefs have not been subjected to selective tests, because they were acquired during a developmental period when their host lacked reflective capacities. 

All of this is heavy lifting at the individual level, however, and it is sobering to admit that AOT scales do not provide accurate measures of the tendency to avoid myside bias. If we want to get at people’s attitudes toward scientific evidence on a contested issue, we actually have to take a domain-specific belief that a person has on the matter, present them with contradictory evidence, and see how they assimilate that contradictory evidence (as some studies have done; see Ditto et al. 2019). You cannot just ask people on a questionnaire whether it is good to pay attention to contradictory evidence.

Ultimately, we all need to rely on the “institutions of rationality” (Rauch 2021) that provide the epistemic tools to deal with what Pinker (2021), aptly channeling the work of (Dan Kahan 2016; Kahan et al. 2017), calls the “Tragedy of the Belief Commons”. Cultural institutions can enforce rules whereby people benefit from rational tools without having to learn the tools themselves. Pinker (2021) describes some institutional reforms within the media and the internet but laments the state of our universities and their “suffocating left-wing monoculture, with its punishment of students and professors who question dogmas on gender, race, culture, genetics, colonialism, and sexual identity and orientation” (p. 313). He describes how “on several occasions correspondents have asked me why they should trust the scientific consensus on climate change, since it comes out of institutions that brook no dissent” (p. 314). In short, the public is coming to know that universities have approved positions on certain topics, and thus is quite rationally reducing its confidence in research that comes out of them. It is thus consistent with the earlier evidence we have reviewed on how myside bias is independent of intelligence and educational status that university faculties—full of cognitive ability and educational attainment—cannot free themselves from myside bias.

## Figures and Tables

**Table 1:** Recommended 13-Item Actively Open-Minded Thinking Scale.

People should always take into consideration evidence that goes against their opinions.	BR
Changing your mind is a sign of weakness. (R)	REF/ALT/BR/OC
I like to think that my actions are motivated by sound reasons.	REASONS
It is important to stick to your opinions even when evidence is brought to bear against them. (R)	BR/OC/BEL_ID
Intuition is the best guide in making decisions. (R)	REF
Considering too many different opinions often leads to muddled thinking. (R)	AMB/ALT/OC
One should disregard evidence that conflicts with your current opinions. (R)	BR/ALT
Coming to decisions quickly is a sign of wisdom. (R)	REF
Allowing oneself to be convinced by a solid opposing argument is a sign of good character.	BR/BEL_ID/OC
If something I think feels right then I am comfortable, whether or not it is true. (R)	REF/TRUTH
A person should always consider new information.	ALT
People should revise their conclusions in response to relevant new information.	BR
Certain opinions are just too important to abandon no matter how good a case can be made against them. (R)	BR

R = reverse-scored. REF = tendency toward reflection. ALT = concern for alternative explanations. BR = belief revision item. AMB = tolerance for ambiguity. OC = tendency toward overconfidence. BEL_ID = belief identification item. REASONS = value placed on thinking motivated by reasons. TRUTH = value placed on truth. Subjects responded on a six-point scale with no neutral point: disagree strongly (1), disagree moderately (2), disagree slightly (3), agree slightly (4), agree moderately (5), agree strongly (6).

## Data Availability

Not applicable.

## Notes

1 Optimal functioning does not result from maximizing cognitive styles. Instead, rationality, plotted against most thinking dispositions, is an inverted U-shaped function. One does not maximize rationality by maximizing the reflectivity/impulsivity dimension, for example, because a person doing so might get lost in interminable pondering and never make a decision. One does not maximize the dimension of belief flexibility either, because such a person might end up with a pathologically unstable personality. Reflectivity and belief flexibility are “good” cognitive styles only in the sense that most people are too low in both dimensions (Baron 2008). Most people would be more rational if they increased their degrees of reflectivity and belief flexibility, but this does not mean that either of these thinking dispositions should always be maximized.

2 Most of the other facets of openness, as measured in scales tapping the Big Five personality dimensions, are not related to AOT, as conceptualized in the rational thinking literature—for example, facets such as openness to fantasy, aesthetics, feelings, and actions.

3 For example, the comprehensive intellectual humility scale developed by Krumrei-Mancuso and Rouse (2016) includes five items measuring belief identification (e.g., “I feel small when others disagree with me on topics that are close to my heart”, reverse-scored), five items measuring belief revision (e.g., “I am open to revising my important beliefs in the face of new information”), six items measuring respect for the opinion of others (e.g., “I can have great respect for someone, even when we don’t see eye-to-eye on important topics”), and six items measuring the avoidance of overconfidence (e.g., “My ideas are usually better than other people’s ideas”). Likewise, the short humility scale studied by Leary et al. (2017) mixes belief revision items (“I reconsider my opinions when presented with new evidence”) with items tapping respect for alternative opinions (“I recognize the value in opinions that are different from my own”).

4 Our characterization of heuristics and biases tasks as implicating decoupling holds only for those instances where the task truly does create a hostile environment (see Stanovich 2018) for a particular subject because the normative response has not been practiced to automaticity. In cases where the normative response has been practiced to an automaticity so great that it dominates the intuitive response, there is no response conflict and cognitive decoupling is not required (see De Neys 2023; Thompson et al. 2018).

5 Most of the criticisms of this study by Baron (2017) would not reverse the direction of the association between polarization and AOT. They would, instead, drive the association toward zero, thus making the Kahan and Corbin (2016) results more consistent with the conclusion drawn in this section.

6 Stanovich (2018) discusses all the complexities involved in pinpointing the cognitive source of the two responses—particularly the classically normative one, which can originate from either System 1 or System 2.
